# Reviewed article: Rossignoli P, Pontarolo R, Fernandez-Llimos F. Variability in public procurement prices for Group 1A drugs in the specialized pharmaceutical component: an observational study, 2013-2022. Epidemiol Serv Saude. 2025:34;e20240733

**DOI:** 10.1590/S2237-96222025v34e20240733.b

**Published:** 2025-09-01

**Authors:** Inês Ribeiro-Vaz

**Affiliations:** 1 Faculdade de Medicina da Universidade do Porto, Porto, Portugal Faculdade de Medicina da Universidade do Porto Porto Portugal

## Editor’s review

## First round comments

Caros autores,

O estudo contribui para a compreensão de evidência do mundo real sobre as disparidades nos preços de aquisição de medicamentos entre o Ministério da Saúde e a Secretaria Estadual de Saúde do Paraná, no Brasil. Esses resultados podem fomentar o debate e a formulação de políticas vocacionadas para a otimização dos gastos em saúde.

O trabalho está bem estruturado e poderá vir a ser publicado, após revisão baseada nos comentários dos revisores.

Recommendation: Minor revision 

